# Prevalence and predictors of adequate treatment of overt hypothyroidism – a population-based study

**DOI:** 10.17179/excli2021-4291

**Published:** 2022-01-06

**Authors:** Julie Lindgård Nielsen, Jesper Karmisholt, Inge Bülow Pedersen, Allan Carlé

**Affiliations:** 1 Department of Endocrinology, Aalborg University Hospital, Aalborg, Denmark; 2 Department of Clinical Medicine, Aalborg University, Aalborg, Denmark

**Keywords:** treatment, hypothyroidism, overt hypothyroidism, population-based study

## Abstract

The aim of this study is to evaluate the adequacy of treatment, and to identify factors influencing treatment of hypothyroidism. Patients newly diagnosed with overt hypothyroidism (*n*=345) were identified via a register linked to a laboratory database. In selected periods with staff available, 165 patients were invited, and 113 (68.5 %) accepted participating in a comprehensive program including blood tests and completion of questionnaires. We performed a longitudinal follow-up on thyroid function tests 10 years after the diagnosis. Time to reach a serum TSH level of 0.2-10 mU/L (termed as clinically acceptable) and biochemical normalization (TSH: 0.2-5.0 mU/L), respectively, were analyzed using Kaplan Meier survival analysis. Predictors for longer duration to reach the normal TSH range were identified using cox proportional hazards regression. Only 67.7 % of the patients were in the euthyroid range on the long term after diagnosis of overt hypothyroidism (2 years: 59.4 %; 10 years: 67.7 %). Median time to the first normal TSH was 8.9 months (95 % CI: 7.6-10.2 months). The factors associated with longer duration until normalization of TSH after multivariate analysis were age (HR 0.79 per 10 years; 95 % CI: 0.66-0.94; *P *= <0.01), smoking (HR 0.47; 95 % CI: 0.26-0.83; *P *= <0.01), serum TSH at diagnosis (HR 0.96 per 10 mU/L; 95 % CI: 0.93-0.99; *P *= 0.02) and BMI (HR 0.96 per kg/m^2^; 95 % CI: 0.91-0.99; *P *= 0.03). A considerable number of hypothyroid patients remained inadequately treated. When treating hypothyroid patients, special attention should be addressed to those patients who never or lately obtain euthyroid status.

## Abbreviations (listed alphabetically)

**BMI** Body Mass Index

**CI ** Confidence Interval

**DanThyr** The Danish Investigation of Iodine Intake and Thyroid Diseases 

**GP** General Practitioner

**HR** Hazard Ratio

**INR** International Normalized Ratio

**IQR** Interquartile Range (25 to 75 % range)

**PPTD** Postpartum Thyroid Dysfunction

**SAT** Subacute Thyroiditis

**SPSS** Statistical Package for Social Sciences 

**TFT** Thyroid Function Test

**TgAb** Thyroglobulin Antibodies 

**TPOAb** Thyroid Peroxidase Antibodies 

**TTR** Time in Therapeutic Range

## Introduction

Thyroid hormone replacement with synthetic levothyroxine is considered the standard of care for treating hypothyroidism (Jonklaas et al., 2014[14]). The therapeutic goals in hypothyroidism are to normalize serum TSH, relieve patients' symptoms, avoid long-term complications, and to avoid over-treatment (Jonklaas et al., 2014[14]). However, inadequate treatment is common in hypothyroid patients with only 43-68 % of the patients reported to be in the euthyroid range (Parle et al., 1993[22]; Canaris et al., 2000[1]; Díez 2003[10]; Somwaru et al., 2009[32]; Okosieme et al., 2011[21]; Vigário et al., 2013[36]; Yavuz et al., 2017[39]).

Suboptimal thyroid hormone replacement may be harmful. Hypothyroidism is associated with increased serum lipids (Canaris et al., 2000[1]; Duntas, 2002[11]), increased psychiatric morbidity (Thvilum et al., 2014[34]), impaired quality of life (Vigário et al., 2013[36]; Winther et al., 2016[38]) as well as loss of labor market income and increased risk of disability pension (Thvilum et al., 2014[35]). Both under- and over-treatment are associated with increased mortality (Walsh et al., 2005[37]; Lillevang-Johansen et al., 2018[18]; Thayakaran et al., 2019[33]), cardiovascular disease (Walsh et al., 2005[37]; Flynn et al., 2010[12]; Rodondi et al., 2010[29]; Lillevang-Johansen et al., 2019[17]), fractures (Flynn et al., 2010; Thayakaran et al., 2019[33]) and cardiac arrhythmias (Sawin et al., 1991[31]; Flynn et al., 2010[12]). A review by Rodondi et al. (2010[29]) comprising a large number of patients (*n*=55,287) showed that subclinical hypothyroidism is associated with an increased risk of coronary heart disease events, including mortality in those with higher TSH levels, particularly in patients with a TSH of 10 mU/L or above (Rodondi et al., 2010[29]). In an analysis of a large database of levothyroxine treated patients (*n*=17,684), Flynn et al. (2010[12]) showed an increased risk of cardiovascular disease, dysrhythmias and fractures in patients with either elevated TSH (>4.0 mU/L) or a suppressed TSH (≤0.03 mU/L). However, in low but detectable TSH concentrations (0.04-0.4 mU/L) no harmful events were registered. A registry-based case-control study by Lillevang-Johansen et al. (2019[17]) (*n*=216,894) showed a dose-response relationship between out-of-target TSH and cardiovascular disease, indicating that the duration of over- or under-treatment of hypothyroidism might be of critical importance. 

The reasons for inadequate treatment vary and may include factors such as poor patient compliance, inappropriate dosage, malabsorption or concurrent use of medications interfering with levothyroxine absorption (Morris, 2009[20]).

To our knowledge, no studies have investigated the length of time it takes to achieve biochemical normalization of thyroid function and which factors may predict the normalization rate. This is relevant in order to set patients' expectations and to identify groups who might need more attention from the treating physician. 

The objective of this study was to evaluate the adequacy of treatment in a 10-year follow-up period after overt hypothyroidism was diagnosed. In addition, we wanted to identify clinical and demographic factors influencing the time needed to reach biochemical normalization of thyroid function after overt hypothyroidism was diagnosed.

## Subjects and Methods

The Danish Investigation of Iodine Intake and Thyroid Diseases (DanThyr) was established in 1997 to monitor the Danish iodine fortification program (Rasmussen et al., 1996[26]; Laurberg et al., 2006[16]). The DanThyr program consists of three main parts including a prospective identification of incident cases of overt thyroid dysfunction in an open population cohort of 311,102 subjects from an area in and around the city of Aalborg in the North of Denmark.

### Patients

A Register Database was linked to the laboratory at Aalborg University Hospital covering the whole cohort area. All subjects with elevated serum TSH (>5.0 mU/L) and low total T4 (<60 nmol/L) were identified by the Register Database as potential new cases of overt hypothyroidism and subsequently, each subject was reviewed to verify or disprove the diagnosis (Pedersen et al., 2002[25]). This was performed by contacting the physician who requested the thyroid function test (TFT) and by reviewing available hospital records and registers. Cases with no information of previous overt thyroid dysfunction were verified as new cases if they met at least one of three criteria:

- Sustained biochemical signs of overt hypothyroidism after at least 3 weeks
- Normalization of thyroid function within 3 weeks due to levothyroxine replacement therapy
- Normal thyroid function after 3 weeks without treatment but with a history suggesting a transient condition of hypothyroidism, such as subacute thyroiditis (SAT) or its asymptomatic variant painless thyroiditis; postpartum thyroid dysfunction (PPTD); medication-induced thyroid dysfunction (amiodarone and lithium); radiation-induced thyroid dysfunction; or surgical manipulation of the thyroid gland (Pedersen et al., 2002[25])

A few cases (*n*=11) did not meet any of these criteria. They were of older age (median age 76.3 years), and all died shortly after the blood test (all within 2 months), before a presumably confirmative test could be performed. These patients were considered to be true hypothyroid cases but were excluded from further analysis in the present study. The process of identification and the various types of evaluation and control performed have been extensively described in a previous study (Pedersen et al., 2002[25]).

As described in detail by Carlé et al. (2006[4]), all incident cases of overt hypothyroidism were classified into subtypes. The two major types were spontaneous, presumably of autoimmune origin (Hashimoto's Thyroiditis) (Carlé et al., 2006[3][4]) (*n*=273); and non-spontaneous (*n*=72). The non-spontaneous entities were further divided into PPTD (*n*=21); SAT (*n*=10); radiation or surgery induced hypothyroidism (*n*=10); amiodarone- and lithium-induced hypothyroidism (*n*=19 and 7, respectively); and congenital hypothyroidism (*n*=5). 

**Figure 1:**
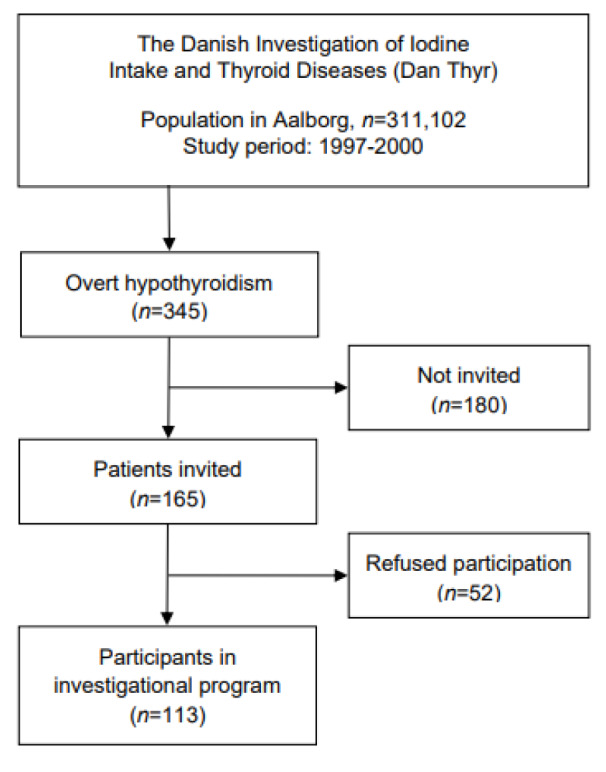
Flowchart depicting the origin and selection of hypothyroid cases (*n*=345) and participants in investigational program (*n*=113) during the study

In the period from March 1997 to December 2000 (1,192,558 person-years of observation), we identified 345 patients with overt hypothyroidism (Figure 1(Fig. 1)). In selected periods with staff available, we invited 165 patients, of whom 113 (68.5 %) gave full participation in a comprehensive investigational program. We only invited patients aged below 80 years.

### Investigational program

As previously outlined in other studies, (Carlé et al., 2014[7], 2015[2], 2016[6]) participants in the investigational program had their height and weight measured, underwent thyroid ultrasound investigation, had thyroid peroxidase antibodies (TPOAb) and thyroglobulin antibodies (TgAb) measured and filled out a comprehensive questionnaire as previously described (Carlé et al., 2014[7], 2015[2], 2016[6]). The questionnaire covered a variety of symptoms and questions on educational level, smoking habits, alcohol consumption and comorbidities (acute myocardial infarction, angina pectoris, cardiac arrhythmia, hypertension, cerebral stroke, epilepsy, diabetes mellitus, asthma, chronic obstructive pulmonary disease, and gastrointestinal ulcer). 

### Thyroid hormone and antibody measurement strategy and analyses 

Blood was drawn at investigation and stored at -20° C until analyzed in random order. For all patients diagnosed with overt hypothyroidism, we registered the specific serum TSH and T4 concentration leading to the diagnosis of overt hypothyroidism. Furthermore, all the subsequent TFTs drawn and analyzed at the Aalborg University Hospital laboratory were imported regularly into the Register Database. In some cases, T3 and T4 were not measured if TSH were within the euthyroid range (0.2 - 5.0 mU/L).

All participating patients had their TPOAb and TgAb measured when visiting our Research Center. In addition, some non-participants had TPOAb measured by their GPs a few weeks after the diagnosis was suspected due to the first TFT. The subjects were considered as antibody-positive, if the antibody concentrations were above the functional sensitivity indicated by the manufacturer (TPOAb+: >30 kU/L, TgAb+: >20 kU/L). Details on TSH, T3, T4, TPOAb and TgAb analyses have been provided in previous publications (Pedersen et al., 2003[24]; Carlé et al., 2006[3]).

### Biochemical thyroid status

We performed a longitudinal follow-up on TFTs from the time of diagnosis and 10 years onwards. We chose 11 reference points with well-defined time intervals: 1 month [½ -1½ months[, 2 months [1½ -2½ months[, 3 months [2½ -4½ months[, 6 months [4½-7½ months[, 9 months [7½ -10½ months[, 12 months [10½ months-1½ y[, 2 years [1½-2½ y[, 3 years [2½-3½ y[, 4 years [3½-4½ y[, 5 years [4½-6 y] and 10 years [9-11 y]. If more than one TFT had been performed during the time interval, we chose the sample performed closest to the reference point.

For all blood samples, thyroid status was classified into one of five groups: overt hypothyroidism (TSH >5.0 mU/L and T4 <60 nmol/L); subclinical hypothyroidism (TSH >5.0 mU/L and T4 ≥60 nmol/L); euthyroidism (TSH 0.2-5.0 mU/L); subclinical hyperthyroidism (TSH <0.2 mU/L, T4 ≤140 nmol/L and T3 ≤2.7 nmol/L); or overt hyperthyroidism (TSH <0.2 mU/L combined with T4 >140 nmol/L and/or T3 >2.7 nmol/L). Furthermore, all blood samples were classified into additionally two clinically relevant groups in terms of avoiding potentially harmful health consequences: one for samples within the clinically acceptable range (TSH 0.2-10.0 mU/L) and one for those outside of this range (TSH <0.2 or >10 mU/L). The lower cut-off level was based on a general recommendation on avoiding thyroid hormone excess due to potential deleterious health effects (Flynn et al., 2010[12]; Jonklaas et al., 2014[14]). The higher cut-off level was chosen in line with various guidelines suggesting that subclinical hypothyroid patients with a serum TSH <10 mU/L may not require adjustment of their thyroid function (Jonklaas et al., 2014[14]; Pearce et al., 2014[23]). Furthermore; a meta-analysis showed that significantly increased risk of cardiovascular mortality and morbidity was primarily observed in individuals with TSH >10 mU/L (Rodondi et al., 2010[29]). 

### Statistical analysis

We used IBM Statistical Package for Social Sciences (SPSS) version 27.0 for calculations and statistical analyses. Baseline characteristics were summarized for participants and non-participants. Medians and interquartile ranges (IQR, 25^th^-75^th^ percentile) were calculated. Time to the first normal and clinically acceptable TSH, respectively, were analyzed using Kaplan Meier survival analysis. Patients who never reached a normal or clinically acceptable TSH, respectively, were censored. Patients who were lost to follow-up, either due to no further measurements or death, were censored at the time of the last TSH measurement (*n=*51). Patients who completed the entire follow-up period without biochemical normalization at any time were censored at the end of the follow-up (*n*=6). 

In the univariate analysis, quantitative variables were divided into subgroups. Median time to the first TSH within the normal range was calculated for all subgroups on all variables. We used Mann-Whitney *U*-test and Kruskal-Wallis test to compare the differences in means. 

A multivariate analysis was carried out using cox proportional hazards regression. The proportional hazard assumption was assessed by calculating the product of time and the variable. If the product of time and the variable were statistically significant, the proportional hazard assumption was violated. No significant deviations from the proportional hazard assumption were found. 

Univariate and multivariate analyses were only conducted on participants in the comprehensive investigational program due to a larger number of variables available on these subjects. 

### Ethical approval

This study was approved by the Regional Ethics Committee in North Jutland. Registry permission was obtained from the Danish Data Protection Agency. All participants provided written informed consent. No conflicts of interest have occurred during implementation or completion of the study. 

## Results

### Baseline characteristics

During the study period, we identified 345 patients with incident overt hypothyroidism. Of these, 113 participated in a comprehensive investigational program. The baseline characteristics of participants (*n*=113) and non-participants (*n*=232) are shown in Table 1(Tab. 1). The participants were on average 17 years younger and were more often women. The participants also had higher serum TSH at diagnosis than non-participants (Table 1(Tab. 1)).

### Control in patients with hypothyroidism

**Figure 2:**
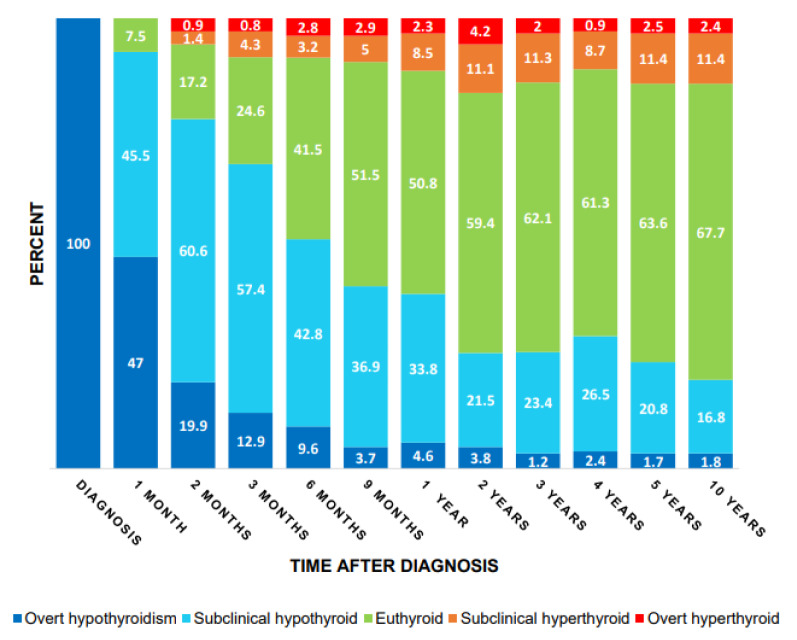
Distribution of thyroid status over time among patients who had a TFT performed within each of the time ranges

Adequacy of therapy was studied for up to 10 years in all 345 patients diagnosed with overt hypothyroidism. During follow-up (mean time: 6.5 years), patients changed biochemical status as shown in Figure 2(Fig. 2). After 10 years of treatment 67.6 % of the patients were in the euthyroid range, while 77.0 % were in the clinically acceptable range (data not shown). Up to 15.3 % were hyperthyroid (subclinical or overt) during the follow-up.

### Normalization of thyroid function

83.5 % of patients (*n*=288) had at least one serum TSH within the normal range (0.2-5.0 mU/L) during the 10-year follow-up. Median time to the first normal TSH was 8.9 months (95 % CI: 7.6-10.2 months). 16.5 % of the patients (*n*=57) never had serum TSH within the normal range during the 10 years follow-up. Among these, 28 patients passed away within the first year after the diagnosis; 6 patients completed the entire follow-up period without any TSH in the normal range at any time; and 23 patients were lost to follow-up before normalization of their thyroid function. Subjects who never became euthyroid were old (median: 73.5 years) and had short follow-up time (mean: 2 years and 1 month). 

**Figure 3:**
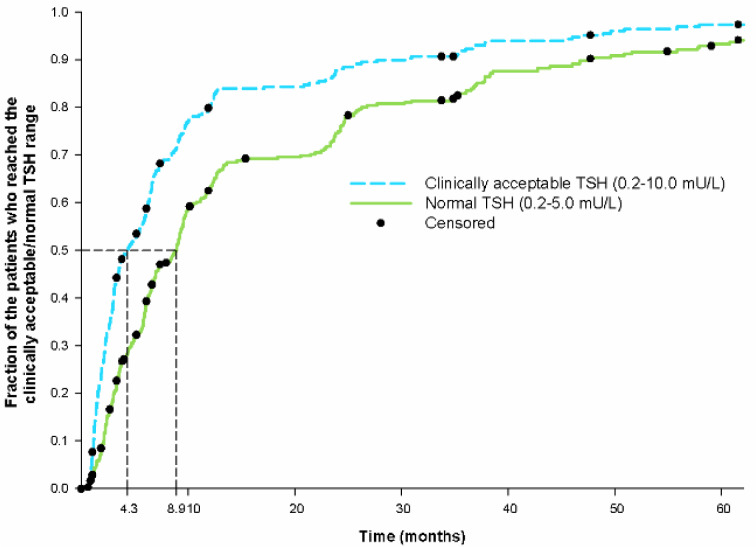
Kaplan Meier curves for the fraction of patients who had a TFT performed within the clinically acceptable and normal TSH range, respectively. Censoring is indicated by the black dot tic mark. For visual purpose the figure does not show the entire follow-up period (11 years). Therefore, few cases are not shown in the figure due to very late occurring normalization. 1 case was left out on the clinically acceptable curve (time 121.9 months). 3 cases were left out on the normal TSH curve (time 117.9, 121.9 and 122.1 months). On the clinically acceptable curve, only the 272 patients who had TSH ≥10 mU/L at diagnosis are included.

We performed an additional analysis to see how many patients reached the acceptable TSH range. While 12.7 % (*n*=44) had a serum TSH of 5-10 mU/L at diagnosis, an additional 78.8 % (*n*=272) had at least one TFT during the follow-up period with TSH within the clinically acceptable range (0.2-10.0 mU/L). Median time to the first TFT within this range was 4.3 months (95 % CI: 3.1-5.5 months). Figure 3(Fig. 3) shows Kaplan Meier curves for the fraction of patients within the normal and clinically acceptable TSH range, respectively.

### Factors influencing the time to normalization of thyroid function after diagnosis of overt hypothyroidism

Time to the first normal TSH according to different clinical and demographic factors are shown in Table 2(Tab. 2). In the univariate analysis, increasing age and spontaneous autoimmune hypothyroidism were associated with longer duration until the first normal TSH. Borderline associations to longer time until normal TSH were high BMI, high serum TSH at diagnosis, comorbidity and current smoking. 

The factors associated with longer duration until the first normal TSH after multivariate analysis were age (HR 0.79 per 10 years; 95 % CI: 0.66-0.94; *P *= <0.01), smoking (HR 0.47; 95 % CI: 0.26-0.83; *P *= <0.01), serum TSH at diagnosis (HR 0.96 per 10 mU/L; 95 % CI: 0.93-0.99; *P *= 0.02) and BMI (HR 0.96 per kg/m^2^; 95 % CI: 0.91-0.99; *P *= 0.03) (Table 3 (Tab. 3)). 

See also the Supplementary data.

## Discussion

### Adequacy of treatment

Our study shows that 10 years after being diagnosed with hypothyroidism, a significant proportion of the patients (32 %) were inadequately treated. A high prevalence of inadequate treatment for hypothyroidism seems to occur worldwide (Parle et al., 1993[22]; Canaris et al., 2000[1]; Díez, 2003[10]; Somwaru et al., 2009[32]; Okosieme et al., 2011[21]; Vigário et al., 2013[36]; Yavuz et al., 2017[39]). Four studies observed prevalence of euthyroidism during treatment similar to our finding (58-68 %) (Canaris et al., 2000[1]; Díez, 2003[10]; Okosieme et al., 2011[21]; Vigário et al., 2013[36]), while other studies observed lower prevalence ranging from 43-55 % in the euthyroid range (Parle et al., 1993[22]; Somwaru et al., 2009[32]; Yavuz et al., 2017[39]). Based on available studies (Parle et al., 1993[22]; Canaris et al., 2000[1]; Díez 2003[10]; Somwaru et al., 2009[32]; Okosieme et al., 2011[21]; Vigário et al., 2013[36]; Yavuz et al., 2017[39]) we calculated an overall prevalence of euthyroidism to be 58.4 % (4,201/7,195). We reported almost identical figure two years after hypothyroidism was diagnosed (59.4 %, *P*=0.10). However, 10 years after diagnosis our patients were more often euthyroid (67.7 %, *P*=0.02).

We found that up to 15 % had low TSH as an indication of over-treatment, which is a similar or a slightly lower prevalence than observed in many other studies (Parle et al., 1993[22]; Canaris et al., 2000[1]; Okosieme et al., 2011[21]; Vigário et al., 2013[36]; Yavuz et al., 2017[39]). In the Cardiovascular Health Study 41 % of thyroid hormone users aged 65 or older were over-treated (Somwaru et al., 2009[32]), while a study conducted on outpatients aged 55 or older in a hospital endocrinology clinic in Spain observed a 5 % prevalence of over-treatment (Díez, 2003[10]). Okosieme and colleagues (2011[21]) found that under-treatment was associated with male gender and younger age, while over-treatment was associated with longer duration of treatment and inversely associated with diabetic comorbidity. In our study, over-treatment also appeared to be associated with longer duration of treatment, as the proportion of over-treated patients increased over time, especially after two years. In keeping with our findings, Diez (2003[10]) found that shorter duration of treatment was associated with inadequate treatment. Although we observed a high prevalence of inadequate treatment, only few patients (2-8 %) had overt thyroid dysfunction after the first 9 months after diagnosis. 

### Time to normalization of thyroid function

We found that the median time to the first TSH within the clinically acceptable range of 0.2-10 mU/L was 4.3 months. This indicates that most patients relatively fast reach a level of thyroid function where the risk of adverse health consequences due to thyroid dysfunction is low (Flynn et al., 2010[12]; Rodondi et al., 2010[29]). However, the median time to the first normal TSH was 8.9 months, indicating that the final adjustment until complete normalization of thyroid function takes longer. 

To our knowledge, no study has investigated which clinical and demographic factors are influencing time to biochemical normalization of thyroid function. However, several studies have investigated factors influencing dose requirements, and there might be a connection between the required dose and the time to normalization of thyroid function. There is consistent evidence that body weight, TSH goal, etiology of hypothyroidism and age influence dose requirements (Jonklaas et al., 2014[14]). 

In our study, the degree of hypothyroidism influenced the time to normalization of thyroid function. Patients with a higher serum TSH at diagnosis reached the normal TSH range later. For every 10 mU/L increase in serum TSH at the time of diagnosis, the likelihood of having a normal TSH was 4 % lower at any given time point, meaning that a patient with a TSH of 100 mU/L at time of diagnosis would have 36 % less chance of having a normal TSH at any given time point compared to a patient with a TSH of 10 mU/L at time of diagnosis. 

We also found that high age at diagnosis was associated with longer duration until normalization of thyroid function. For every 10 years age, the likelihood of having normal TSH was 21 % lower at any given time point after diagnosis. This slower rate of normalization might be intentional as a result of the “start low and go slow” recommendation in older people (Jonklaas et al., 2014[14]). One could expect that the presence of comorbidity would influence the time to normalization due to the fact that patients with ischemic heart disease should be started on low levothyroxine dose with gradual increases (Chakera et al., 2012[8]). This was not the case in our study, which might be explained by the lack of distinction between cardiovascular and non-cardiovascular comorbidity due to small numbers in each group. 

Smoking was also associated with longer duration until normalization of thyroid function with half as many smokers being euthyroid at any given time point. This significant role of smoking in hypothyroidism is not as well established as in Graves' disease (Sawicka-Gutaj et al., 2014[30]).

High BMI was associated with longer duration until normalization of TSH. For every kg/m^2^, the likelihood of having a normal TSH was 4 % lower at any given point. These data indicate that levothyroxine dose higher than 1.6 μg/kg (Jonklaas et al., 2014[14]) may be expected when treating obese overtly hypothyroid patients.

### Treatment adequacy of other diseases 

When comparing treatment of hypothyroidism with treatment of other diseases within the field of internal medicine rather comparable measures were obtained. Warfarin anticoagulation in patients with atrial fibrillation is by the Danish Society of Cardiology considered of good quality when time in therapeutic range (an international normalized ratio [INR] between 2-3) (TTR) is at least 70 % (Grove et al., 2021[13]). In hypertension, data indicates that only 39 % of patients manage to achieve an adequate level of blood pressure control (Redon et al., 2016[27]). A Danish diabetes treatment report stated that only 73 % of all hospital out-patients with type 2 diabetes reached the predefined HbA1c goal below 70 mmol/mol. Here, large regional differences were reported with a range between 62-76 % adequately treated (RKKP, 2020[28]). Taken into account that serum TSH may vary up 40 % between two tests without reflecting a true difference (Karmisholt et al., 2008[15]), our results of 68 % within the normal TSH range and 77 % reaching a clinically acceptable TSH level is conceivably an acceptable level.

### Study strengths and limitations 

A strength of this study is the prospective inclusion of overt hypothyroid patients as they were diagnosed in the population. Hence, the present study is free of hospital referral bias, which has previously been shown to lead to overrepresentation of patients who are younger and marginally more hypothyroid (Carlé et al., 2009[5]). However, not all patients were invited to participate in the comprehensive investigational program, and we chose not to invite any patients above the age of 80, which accounted for 14 % of patients newly diagnosed with overt hypothyroidism. Thus, some degree of selection bias may still be present. 

Additionally, a strength of this study is the long follow-up time on TFTs (10 years). Most studies (Parle et al., 1993[22]; Canaris et al., 2000[1]; Somwaru et al., 2009[32]; Okosieme et al., 2011[21]; Vigário et al., 2013[36]; Yavuz et al., 2017[39]) only present data on a specific given time. Our study demonstrated that the timing of follow-up is of great significance for the results. The proportion of inadequately treated patients showed considerable variation during the first 2 years after diagnosis, while it did not seem to change much between 2 to 10 years of treatment. Another strength is the relatively high number of patients included. Also, we have a large amount of data on the 113 participating patients. However, we only have information on this data on the specific time they participated in the comprehensive investigational program, and some variables such as comorbidity, smoking habits and BMI might change over time. Therefore, these variables must be considered with caution when applied on blood samples taken years after diagnosis. 

An important limitation of the study is that we do not have any information on treatment (preparation, dose, dose corrections and compliance). Also, we do not have any information on other medications being taken that could influence the absorption of levothyroxine.

An important consideration is that patients were diagnosed with hypothyroidism between 1997 and 2000. Since then, there has been much debate on treatment of hypothyroidism and patients' satisfaction. New treatment regimens have been added and several substitution preparations are now available (Chiovato et al., 2019[9]). Furthermore, there has been a considerable decrease in the threshold for initiating levothyroxine therapy in hypothyroid patients in Denmark in 2001-2015 (Medici et al., 2019[19]). Thus, our study results may not completely reflect how substitution therapy is performed in 2021. Further studies may reveal whether hypothyroid patients are treated better today than decades ago. 

## Conclusion

A considerable number of hypothyroid patients remain inadequately treated with only 59-68 % in the euthyroid range 2-10 years after diagnosis of overt hypothyroidism. 50 % of the overt hypothyroid patients achieved biochemical normalization of their thyroid function within 9 months after the diagnosis. When treating hypothyroid patients, physicians should pay more attention to patients with high age, high TSH at diagnosis, high BMI and smokers as our results suggest that these groups achieve biochemical normalization at a slower rate. 

## Declaration

### Declaration of interest

The authors declare no conflicts of interest that may be perceived as prejudicing the impartiality of the research reported.

### Funding

This study was part of DanThyr, and it was supported by the following grants: IMK General Foundation; The Danish Council for Independent Research; Ministry of Food, Agriculture and Fisheries; the Danish Agency for Science, Technology and Innovation, Institute for Clinical Medicine, University of Aarhus; and Aase og Ejnar Danielsens Foundation.

### Acknowledgments

We are indebted to the general practitioners in Copenhagen and Northern Jutland, and to clinical chemical laboratories at Aalborg Hospital, Bispebjerg Hospital, Frederiksberg Hospital, as well as Laboratory of General Practitioners in Copenhagen for their helpful collaboration in identifying patients.

## Supplementary Material

Supplementary data

## Figures and Tables

**Table 1:**
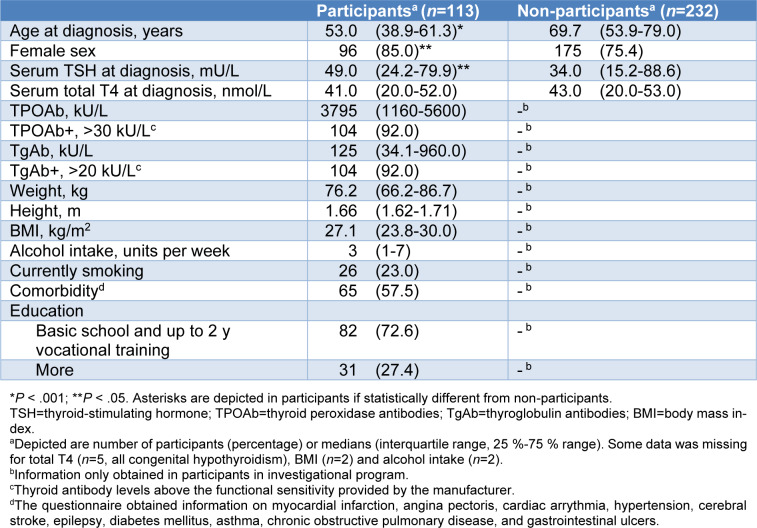
Baseline characteristics of participants in the investigational program and the non-participants

**Table 2:**
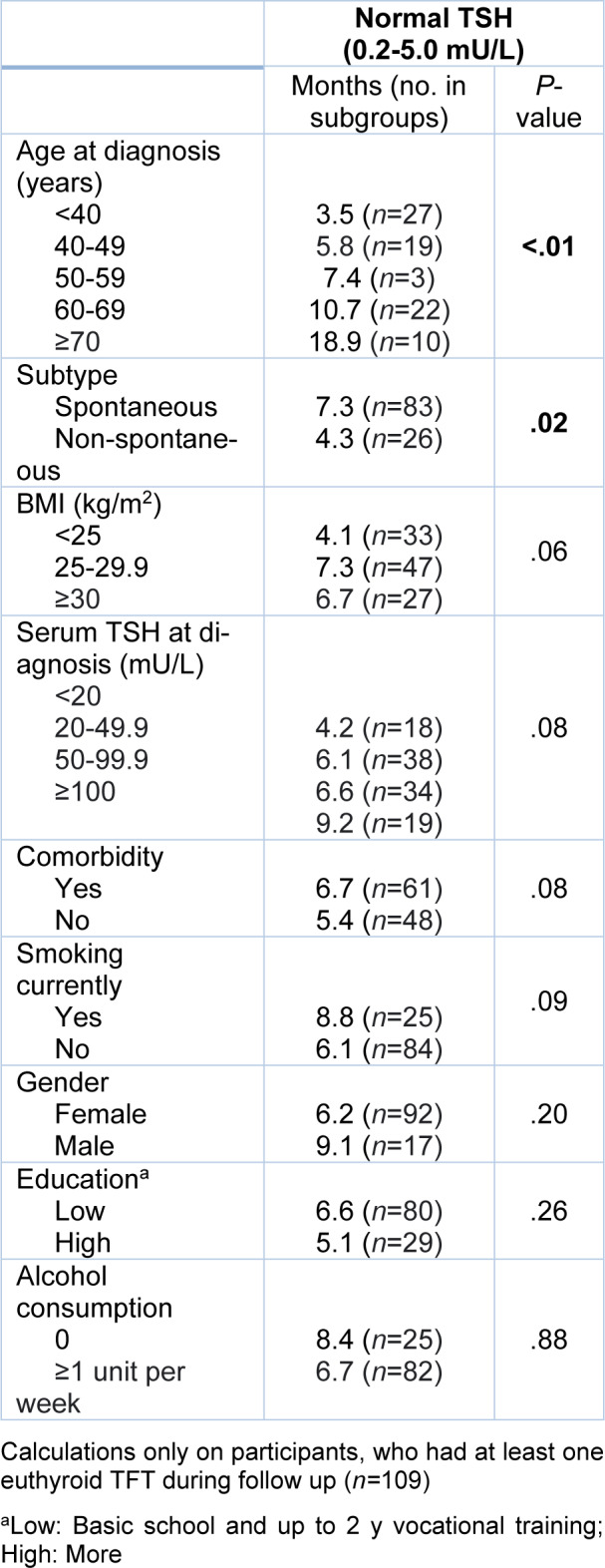
Univariate analysis showing median months to first normal TSH among subgroups within various clinical and demographic variables

**Table 3:**
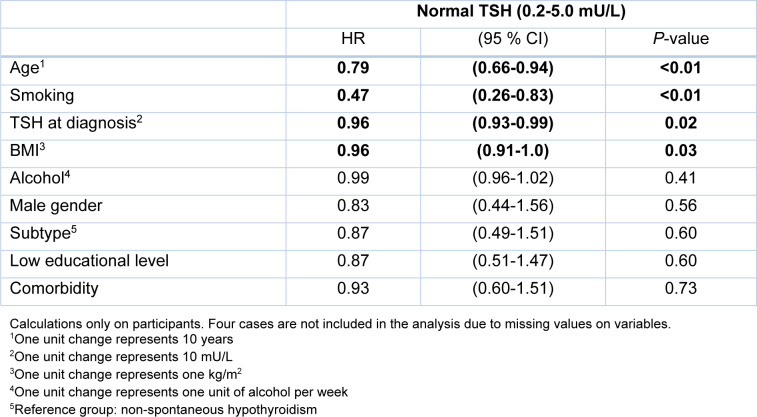
Multivariate analysis with cox proportional hazards regression, showing hazard ratios (HRs) with 95 % confidence intervals (CI) for first TSH within normal range according to clinical and demographic factors
